# Analysis of bleeding after ultrasound-guided needle biopsy of benign cervical lymph nodes

**DOI:** 10.1186/s12893-023-01964-1

**Published:** 2023-03-29

**Authors:** Wenzhi Zhang, Gaoyi Yang, Jianping Xu, Tu Ni, Wei Tang, Meiling Zhou

**Affiliations:** 1 Department of Ultrasonography，Affiliated Hangzhou Chest Hospital, Zhejiang University School of Medicine（Integrated Chinese and Western Hospital of Zhejiang Province, Hangzhou Red Cross Hospital)）, hangzhou, Zhejiang China No.208 Huancheng East Road, Hangzhou, Zhejiang,; 2 Department of Pathology, Affiliated Hangzhou Chest Hospital, Zhejiang University School of Medicine (Integrated Chinese and Western Hospital of Zhejiang Province, Hangzhou Red Cross Hospital), No.208 Huancheng East Road, 310003 Hangzhou, Zhejiang China

**Keywords:** Ultrasound, Cervical, Lymph nodes, Needle biopsy, Bleeding

## Abstract

**Aim:**

Summarized the incidence of bleeding after ultrasound-guided coarse needle biopsy (US-CNB) of benign cervical lymph nodes.

**Methods:**

We retrospectively examined the clinical and follow-up records of 590 patients with benign cervical lymph node disease who underwent US-CNB at our hospital during February 2015–July 2022 and were confirmed to have the disease by CNB and surgical pathology. The number of cases, types of diseases, and degree of bleeding of all patients with bleeding after US-CNB were statistically analyzed.

**Results:**

Of the 590 patients, bleeding was noted in 44 cases(7.46%), and the infectious lymph node bleeding rate was 9.48%. Infectious lymph nodes were more likely to bleed than noninfectious lymph nodes after CNB, ,*x*^2^ = 8.771; *P* = 0.003, Lymph nodes with pus were more likely to bleed than solid lymph nodes after CNB, *x*^2^ = 4.414; *P* = 0.036,.

**Conclusion:**

The bleeding of all patients after CNB was minor bleeding. Infected lymph nodes bleed more frequently than noninfected lymph nodes. Lymph nodes with mobility and a large pus cavity, are more likely to bleed after CNB.

## Background

With an increasing awareness regarding health among the people, the number of individuals diagnosed with cervical lymphadenopathy during physical examination has been increasing. Lymph node tuberculosis, reactive hyperplasia, necrotizing lymphadenitis, lymphomas, and cancer metastasis are the common causes of cervical lymphadenopathy. However, determining the nature of enlarged lymph nodes is critical for subsequent treatment [1, 2]. The traditional diagnostic method is pathological examination after surgical biopsy, which is the gold standard for diagnosing lymphadenopathy; however, it is a highly invasive approach, which can damage the healthy tissues, and the patients are at high-risk of sustaining injuries. Ultrasound-guided coarse needle biopsy (US-CNB) of the cervical lymph nodes is faster, safer, and requires a shorter recovery time than the traditional surgical biopsy approach; it is widely accepted by clinical physicians and patients. Several studies have reported that the success rates of surgical CNB and postoperative pathological diagnosis are high [3–6]. However, only a few studies have reported on the bleeding rate after CNB of benign cervical lymph nodes. Thus, this study determined the incidence of bleeding during and after CNB of cervical benign lymph nodes to provide a reference for preoperative evaluation of cervical lymph node CNB.

## Materials and methods

### Patient selection

The Medical Ethics Committee of Affiliated Hangzhou Chest Hospital of Zhejiang University reviewed and approved this study; the patients who participated provided informed consent. All methods were conducted in accordance with the relevant guidelines and regulations or declaration of Helsinki. In this study, 590 patients with benign cervical lymph nodes confirmed by pathology after US-CNB or surgery who were admitted to our hospital during February 2015–July 2022 were included and retrospectively analyzed.

Bleeding during and after US-CNB was determined by US exploration as an anechoic or hyperechoic zone around and inside the lymph nodes as follows: (1) Minor bleeding: (a) crescent-shaped bleeding around lymph nodes and (b) lymph node bleeding, with no significant increase in lymph node volume compared to before CNB. (2) Moderate bleeding: (a) bleeding in the lymph nodes, with the volume of lymph nodes higher than that before CNB; (b) extensive perilymph node bleeding and compression, lymph node displacement, deformation, and even surgical intervention. (3) Massive bleeding: bleeding inside and around lymph nodes, lymph nodes apparently surrounded by hematoma, or lymph nodes squeezed and displaced by hematoma as detected on ultrasound scan, necessitating surgical treatment. The degree of bleeding in all patients was evaluated by three attending physicians with 5 years of experience in lymph node CNB. The inclusion criteria were as follows: (a) patients with cervical lymph node enlargement with the maximum short diameter of lymph nodes > 0.5 cm and length to diameter > 1.0 cm; (b) those aged > 18 years; (c) those without serious cardiopulmonary dysfunction; (d) those who could tolerate needle biopsy; and (e) those whose blood clotting function was normal.

The exclusion criteria were as follows: (a) patients with a history of taking food allergy medications to reduce allergic and hypersensitivity reactions during SonoVue use; (b) those who took anticoagulant drugs in the previous week; (c) those with a history of mental illness who could not cooperate with needle biopsy; (d) those with skin damage, skin ulcer, and severe psoriasis at the puncture site; and (e) those whose target lymph nodes were located around blood vessels and could not be routinely sampled or those who were assessed preoperatively for severe bleeding and other complications after sampling.

### US and CEUS examinations

In this study, the instrument used was Philips iU22® US machine (Philips, Amsterdam, The Netherlands) with an L12-5 probe and a frequency of 5.0–12.0 MHz; the L9-3 probe had a frequency of 3–9 MHz. The patient was placed in the supine position with the neck fully exposed, and the distribution, size, range of motion, internal echo, and rupture of the lymph node capsule of the cervical lymph nodes were examined using routine US, paying particular attention to the relationship between the lymph nodes and cervical great vessels.

Moreover, SonoVue was used. Before use, 5 mL of normal saline was diluted and shaken well, and 4.8 mL of solution was injected by a projectile through the superficial vein of the elbow; subsequently, 5 mL of normal saline was injected into the flushing tube. In the process of imaging, we used pulsed reverse harmonic imaging with a low mechanical index (0.06). By pressing the time and dynamic storage keys simultaneously with contrast agent injection, the perfusion of the contrast agent to the entire target lymph node was observed. The image obtained from the entire imaging process was saved on the instrument’s hard disk.

### US-CNB

According to the CEUS evaluation, the largest or abnormal lymph node was preferentially selected as the target lymph node. A semi-automatic biopsy gun was used for CNB (18 G, TSK, Japan). Biopsy was performed for all target cervical lymph nodes using core needles under US guidance. After local anesthesia with 2% lidocaine, biopsy needles were passed through the skin to the lymph nodes, and samples were collected; further, three needles were inserted from different directions in the lymph nodes. Two pathological specimens were fixed with 10% formaldehyde and sent for pathological examination, whereas one specimen was placed in a sterile container and sent for bacterial and fungal culture. Intraoperative and postoperative echogenic changes in and around the lymph nodes were observed to determine the degree of bleeding and type of lymph node (anechoic or hyperechoic). To stop the bleeding, postoperative compression was applied for 15–20 minutes. After 1 hour of the operation, a routine US was performed to observe and evaluate the degree of bleeding in and around the lymph nodes. Post-lymph node puncture biopsy bleeding was defined as the presence of anechoic or hyperechoic lymph nodes during and after the operation. Routine US, CEUS, and US-CNB were performed by attending physicians with 5 years of experience in US-CNB of cervical lymph nodes to reduce subjective error.

### Statistical analysis

The Statistical Package for the Social Sciences, version 19.0, was used for data analysis (IBM Corp., USA). The chi-square and Fisher’s exact tests were used to determine the number of patients with bleeding after cervical benign lymph node CNB and the type of disease. Statistical significance was denoted by *P* values of < 0.05.

## Results

### Patient clinical and US features

Of 590 participants, 213 were men and 377 were women, and their average age was 35.71 ± 10.50 (range, 18–63) years. Table 1 shows the patients’ clinical symptoms and the CEUS pattern of the target lymph nodes. Reactive hyperplasia, necrotizing lymphadenitis, Castleman’s disease, Kimura’s disease (KD), sarcoidosis, lymph node tuberculosis, nontuberculous mycobacterial lymphadenitis, common bacterial lymphadenitis, fungal lymphadenitis, and viral lymphadenitis were among the benign lymph node diseases identified in the participants. Table 2 shows the types of benign lymph node diseases and the number of cases of bleeding after needle biopsy. Overall, 4 (9.09%) and 40 (90.90%) cases were without and with infection, respectively. When the two groups were compared, *x*^2^ was 8.771 and *P* was 0.003, indicating that the difference was statistically significant.

**Table 1:** The age distribution, lymph node distribution, and lymph node CEUS mode.

	Cases(n)	Percentage (%)
Lymph node distribution		
Bilateral	407	68.98
Unilateral	183	31.01
Amount		
Single	43	7.28
Multiple	547	92.71
Pain and tenderness		
Yes	175	29.66
No	415	70.34
Motion of lymph node		
Movable	155	26.27
Not movable	435	73.73
History of cancer		
Yes	63	10.68
No	527	89.32
CEUS findings		
Homogeneous enhancement	161	27.28
Heterogeneous enhancement	429	72.71
CEUS mode		
Centripetal enhancement	16	2.71
Diffuse enhancement	123	20.81
Enhancement from the lymphatic hilum to the periphery	451	76.44

Note: CEUS, contrast-enhanced US

**Table 2:** Benign cervical lymph node diseases and bleeding after CNB.

Disease categories	Cases(n)	Bleeding cases(n)	Bleeding rate(%)
Reactive hyperplasia of lymph nodes	110	3	2.73
Infected lymph nodes (422)		40	9.48
Common bacterial infectious lymphadenitis	33	3	9.09
lymph node tuberculous	357	36	10.08
Non-tuberculous mycobacteria	18	1	5.56
Fungal infective lymphadenitis	5	0	0
Viral lymphadenitis	9	0	0
Rare lymph node disease (58)		1	1.72
Sarcoidosis	15	0	0
Castleman disease	8	1	12.5
Necrotizing lymphadenitis	33	0	0
Kimura’s disease	2	0	0
Total	590	44	7.46%

Note: The bleeding rate after needle biopsy of infectious lymphadenitis was compared with that after CNB of benign non-infectious lymph nodes; the *x*^2^ value was 8.771, and the *P* -value was 0.003, which was statistically significant.

**Fig. 1:**
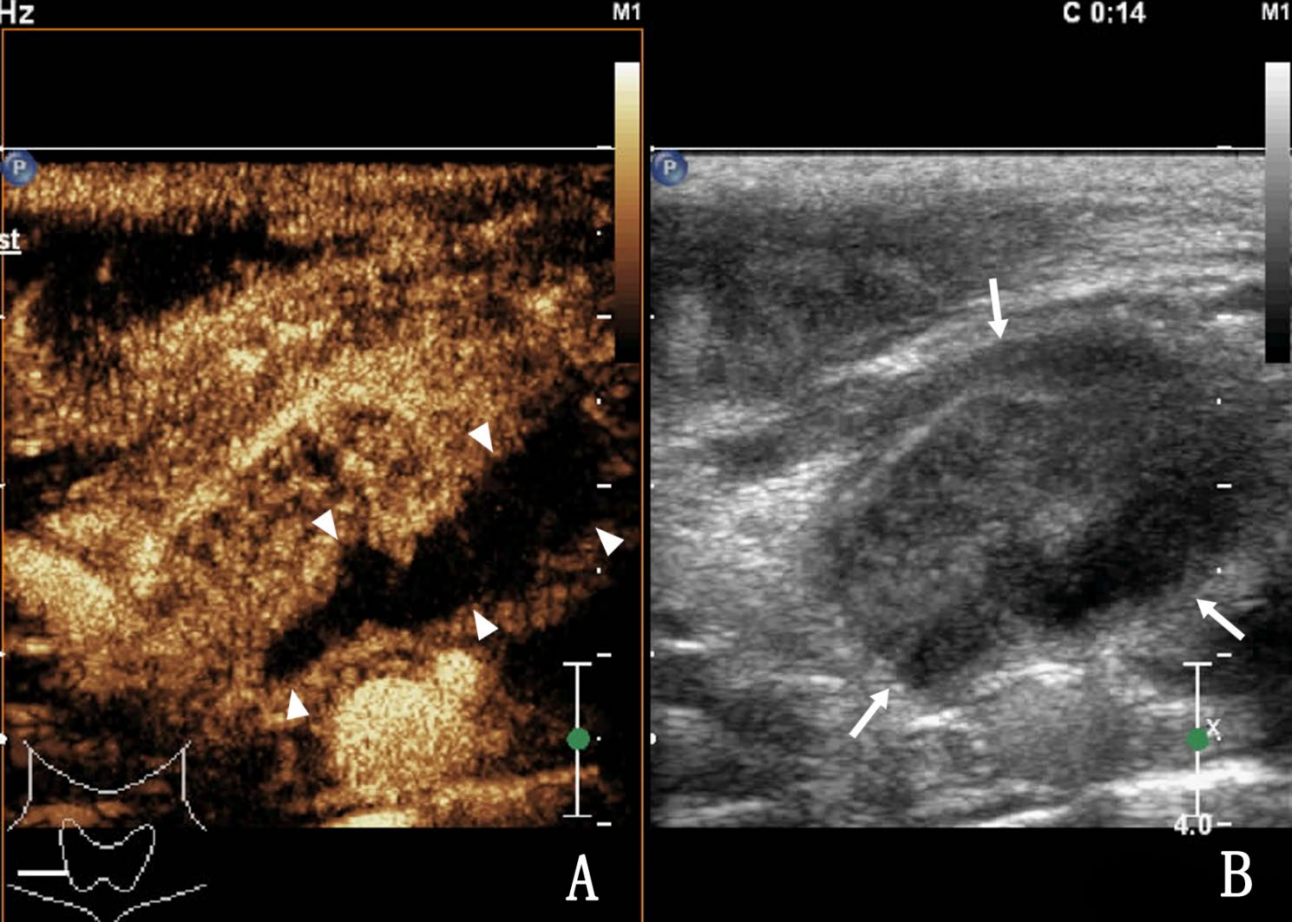
A 31-year-old woman with right cervical lymph node tuberculous. A, CEUS revealed heterogeneous enhancement in lymph nodes, with irregular areas without enhancement, indicating liquefied necrosis (triangle arrow). B, Routine US control view; the arrow shows the diseased lymph node, indicating the target lymph node for needle biopsy.

**Fig. 2:**
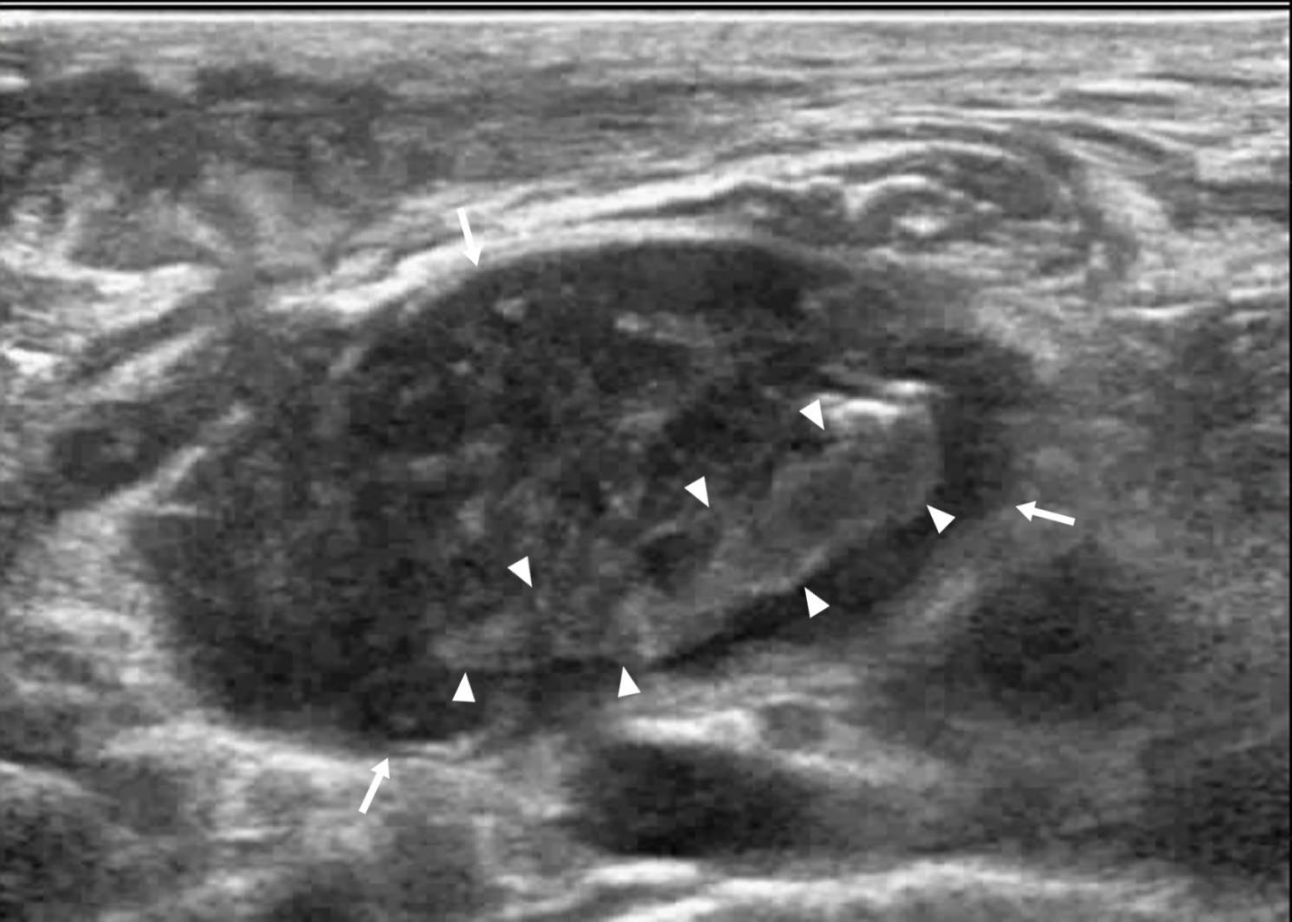
The same patient as in Fig. 1. The arrow shows the target lymph node. At the end of the first needle biopsy, the non-enhanced area of the lymph node changed from preoperative anechoic to hyperechoic during the second needle biopsy, demonstrating minor bleeding in the lymph node (triangle arrow).

**Fig. 3:**
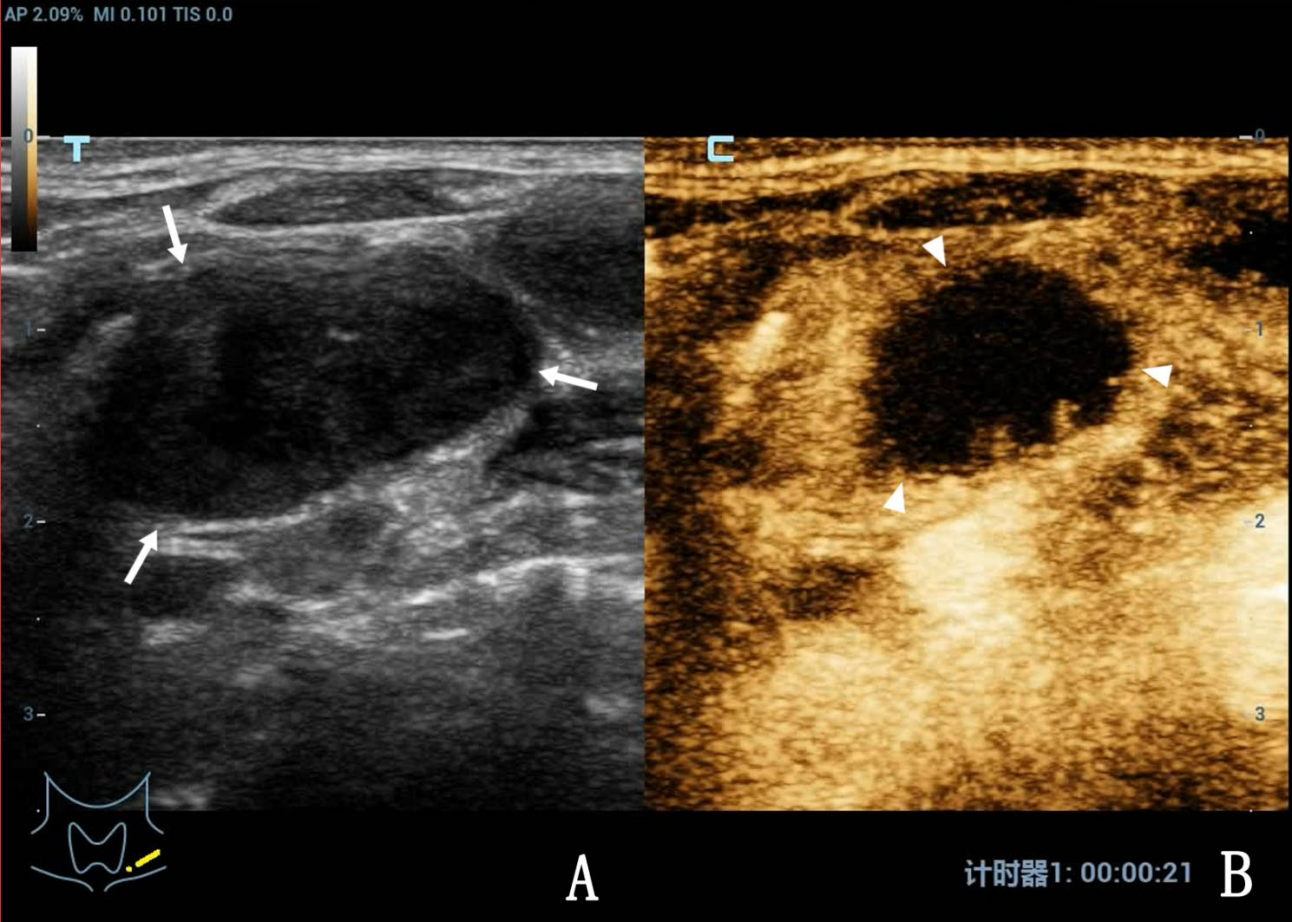
A 27-year-old woman with left cervical lymph node tuberculosis. A, Routine US revealed hypoechoic lymph nodes (arrow), the disappearance of the lymph hilus, and a clear capsule. B, CEUS results revealed heterogeneous enhancement of the diseased lymph nodes, with large areas without enhancement, also known as the abscess cavity (triangle arrow), and a septal enhancement was observed in the cavity.

**Fig. 4:**
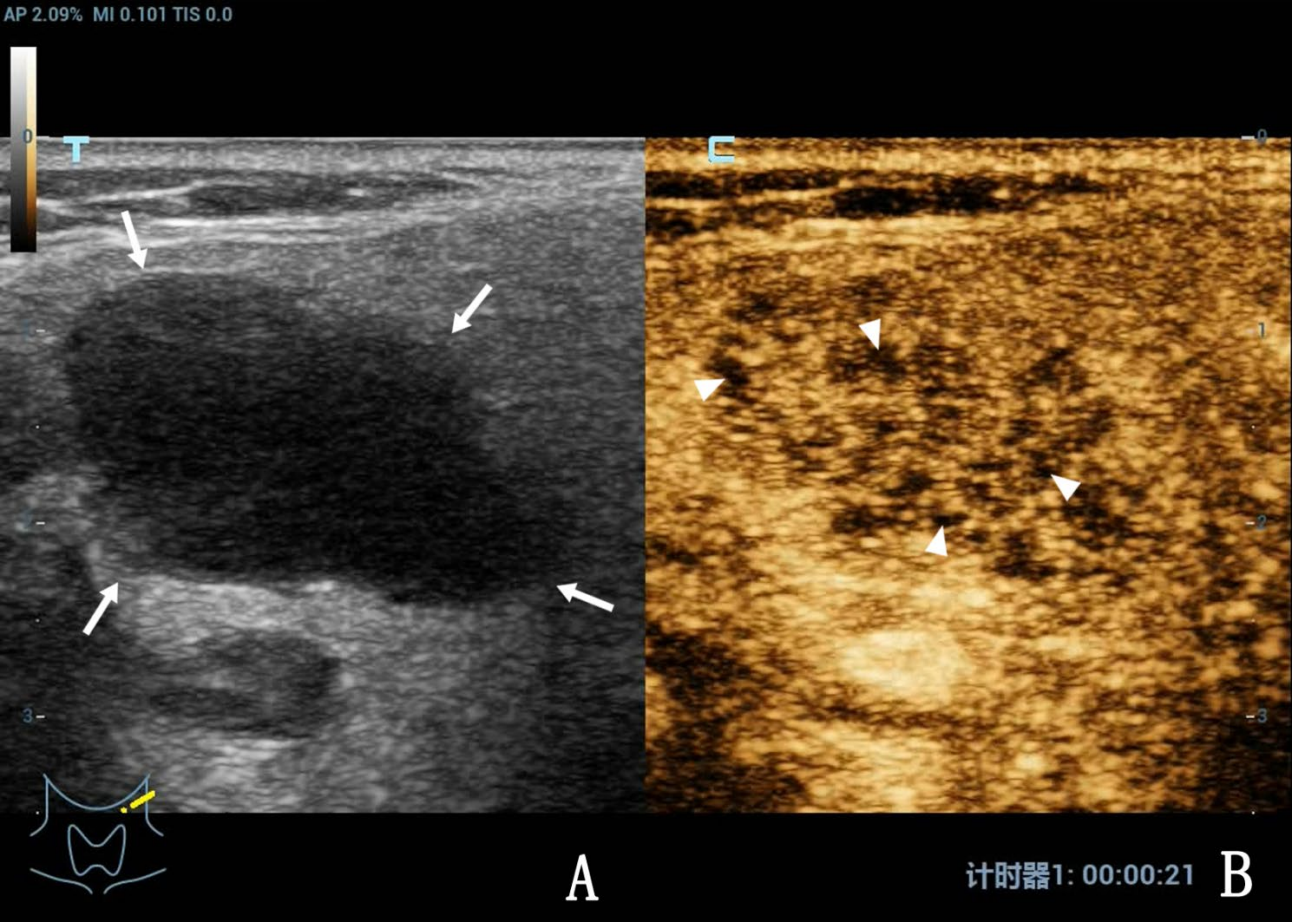
A 24-year-old woman with left cervical lymph node tuberculosis. A, Routine US revealed that the diseased lymph nodes were hypoechoic (arrow), the lymph hilum disappeared, and the boundary between the local lymph nodes and surrounding tissues was unclear. B, CEUS revealed heterogeneous enhancement of the diseased lymph nodes, with diffuse punctured areas without enhancement observed inside the nodes, also known as the tiny abscess cavity (triangle arrow). **Permission to Reuse and Copyright**

Of 44 patients with intra-lymph node and peripheral bleeding after CNB (Figs. 1 and 2), 4 had perilymph node bleeding, and the remaining patients had intralymph node bleeding. Preoperative palpation revealed that the lymph nodes were mobile and immobile in 6 (13.36%) and 38 (86.36%) cases, respectively. CEUS results revealed heterogeneous enhancement, large non-enhanced areas (pus cavity), and punctate region without enhancement in 40, 36 (Fig. 3), and 4 (Fig. 4) cases, respectively. All 44 patients had minor bleeding, and no patient had a large hematoma or bleeding after CNB that necessitated surgical intervention. Forty patients with infected lymph nodes experienced bleeding during and after CNB. Preoperative palpation revealed that lymph nodes were mobile in 2 cases but not in the other 38. Among the 422 patients with infected lymph nodes after CNB, 2 exhibited a sinus tract, both of whom had lymph node tuberculosis.

Lymph nodes with pus cavities (heterogeneous enhancement) bled more frequently than solid lymph nodes (homogeneous enhancement; Table 3).

**Table 3:** Comparison of heterogeneous enhancement of lymph nodes (with pus cavity) and homogeneous enhanced enhancement of lymph node bleeding after CNB.

CEUS findings	Bleeding(n)	No bleeding(n)	%
Heterogeneous enhancement(429)	37	392	8.62
Homogeneous enhancement(161)	6	155	3.72

Comparison of two groups, *x*^2^ was 4.414, and the *P* was 0.036, which was statistically significant.

## Discussion

Lymph node reactive hyperplasia, necrotizing lymphadenitis, lymph node tuberculosis, Castleman’s disease, KD, sarcoidosis, and common bacterial infectious lymphadenitis are some examples of common benign cervical lymph nodes. The most common causes of benign cervical lymph nodes in patients aged 10–50 years are reactive hyperplasia and lymph node tuberculosis. In individuals aged > 50 years, incidence of metastatic lymph nodes is higher than that of tuberculosis and reactive lymphadenopathy [7]. The average age of the patients in this study was 35.71 ± 10.50 years, and most patients were young and middle-aged; this finding was consistent with that of the previous study. Notably, the gold standard for defining cervical lymph node diseases is pathological diagnosis. Owing to the advantages of being minimally invasive, quick, and accurate, US-CNB is widely used in clinical practice. Recent studies [8, 9] have confirmed that CEUS-guided CNB can improve the integrity of sampling and the positive rate of pathological diagnosis in cervical lymph nodes. Another study reported no statistically significant difference in sensitivity between CNB and excisional biopsy [10].

US-CNB is an effective diagnostic tool [11, 12], and some small-scale studies have confirmed its safety in diagnosing cervical lymph node diseases [13–15]. However, only a few studies have reported serious complications following CNB. The incidence of bleeding after CNB was 0.8–3.0% when the average number of CNBs was 2 [16–18]. In this study, the bleeding rate after CNB of benign cervical lymph nodes was 7.46%. This large deviation from previous reports is because before the operation, specimens were planned to meet the needs of routine pathology and immunohistochemistry as well as gene testing and biological culture after CNB. Thus, the number of CNBs in ​​this study was 3, whereas the number of CNBs in previous reports was 2.

The incidence of bleeding from reactive lymph node hyperplasia was low in this study (~ 2.73%); three patients with reactive lymph node hyperplasia experienced bleeding following CNB. This may be because postoperative compression hemostasis point failed to adequately cover the puncture needle tract and the puncture point of the lymph node capsule. Postoperatively, the lymph node activity was relatively high and the puncture point of the lymph node capsule was not effectively compressed, resulting in the puncture point of the lymph node capsule being free from the compression point; however, all cases had minor bleeding. Therefore, we believe that in patients with suspected reactive lymph node hyperplasia, lymph node mobility should be fully evaluated after CNB, and the compression hemostatic point should cover both the needle tract and the area of possible mobility in lymph nodes to effectively prevent bleeding at the entry point of the lymph node capsule.

In this study, the rate of infectious lymph node bleeding was 9.48%, with all cases of minor bleeding. The rate of lymph node tuberculosis bleeding was the highest (~ 10.08%), followed by that of common bacterial lymph node bleeding (9.09%). This may be attributed to the ease with which pus cavities form as well as internal congestion and telangiectasia in infected lymph nodes [19]. Lymph node tuberculosis is characterized by heterogeneous enhancement [8]. In this study, tuberculous lymph nodes with a large nonenhancement range were more likely to bleed than those with punctate nonenhancement; all of these were intra-lymph node bleeding with no perilymph node bleeding associated with needle tract bleeding spilled into the abscess cavity along the needle tract after CNB. Most infectious cases in this study were of lymph node tuberculosis—a chronic infection that frequently causes adhesion between surrounding tissues and lymph nodes and poor palpation activity [20]. Bleeding after lymph node tuberculosis CNB primarily affects lymph nodes. We believe that in infected lymph nodes, patients with good lymph node mobility are more likely to exhibit bleeding around the lymph nodes during and after CNB than those with poor lymph node mobility. This viewpoint needs to be validated by increasing the number of cases of infectious lymph node diseases.

In this study, the incidence of intraoperative and postoperative bleeding was lower in rare lymph node diseases than in reactive lymph node hyperplasia and infectious lymph nodes. We believe that this is due to poor lymph node mobility in rare lymph node diseases, edema of the tissue surrounding the lymph nodes in sarcoidosis and necrotizing lymphadenitis, and adhesion to lymph nodes in necrotizing lymphadenitis [21, 22]. Approximately 90% of KD cases had perilymph node infiltration [23, 24], which is characterized by a hyperechoic ring around the lymph node and borderline and mixed color blood flow signals on US scan. Because of the adhesion between lymph nodes and the surrounding tissues, closed space forms around the lymph nodes. Bleeding after CNB is primarily caused by the needle tract overflow. Postoperative hemostasis and compression can help prevent postoperative bleeding. However, in Castleman’s disease, the lymph nodes are frequently large, and there is some pressure between the lymph nodes and surrounding tissues. Postoperative bleeding can be effectively reduced with compression hemostasis after CNB. The number of rare lymph nodes in this study was limited, which must be confirmed by increasing the sample size or conducting a multicenter study.

According to some studies [25], owing to the presence of small lymph nodes, CNB needles usually obtain samples before reaching the capsule, along with the outer connective tissue, lymph node capsule, and lymph tissue, which may a reason for bleeding after CNB.

A sinus tract can be formed in patients with infected lymph nodes after CNB, and further studies are required to determine whether there is a link between sinus tract formation and bleeding. However, sinus tract formation is linked to the operator’s manipulation and the pathological features of lymph node tuberculosis. We believe that the surgeon of patients with infected lymph nodes should master the skills to ensure the following during CNB: (a) The puncture point should avoid raised, red, or broken areas of skin; (b) To ensure the safety of CNB operation, the surgeon should try choosing the highest point of the body position into the needle; and (c) If the CEUS evaluation reveals large cavities of pus in the lymph nodes, the surgeon should attempt to drain the pus before compression and induce hemostasis.

This study has some limitations. First, this was a single-center study, necessitating multicenter data to confirm its accuracy. Second, we a performed retrospective analysis, necessitating prospective confirmatory studies. Finally, only benign lymph node diseases were evaluated in this study to summarize the bleeding risk of all lymph node diseases.

## Conclusion

US-CNB is commonly used to diagnose benign cervical lymph node diseases. Although the bleeding rate was 7.46% in this study, all cases were of minor bleeding, primarily affecting lymph nodes. Thus, US-CNB was idenftied as a safe and effective minimally invasive diagnostic method. Given the requirement of mastering the indications for CNB, infectious lymph nodes in benign lymph node disease are at a higher risk of bleeding. Lymph nodes with pus cavities are more likely to cause intraoperative and postoperative lymph node bleeding than those without pus cavities. Furthermore, in benign lymph node disease, lymph node with poor lymph node mobility have a lower risk of bleeding during and after CNB than those with good lymph node mobility.

## Data Availability

The raw data supporting the conclusions of this article will be made available by the authors, without undue reservation. For data on this study, please contact the corresponding author.

## List of Abbreviations

CEUS: contrast-enhanced US
KD: Kimura’s disease
US-CNB: ultrasound-guided coarse needle biopsy
